# Prosthetic Rehabilitation of a Patient with an Ocular Defect: A Simplified Approach

**DOI:** 10.1155/2013/207634

**Published:** 2013-02-28

**Authors:** Shivakumar Puranik, Anoop Jain, Sunil Ronad, Sachhi Ramesh, Jagadeesh MS, Puttaraj Kattimani

**Affiliations:** ^1^Department of Prosthodontics, HKE'S S N Institute of Dental Sciences & Research, Gulbarga, Karnataka 585105, India; ^2^Department of Prosthodontics, VP Dental College, Sangli, Maharashtra 416414, India; ^3^Department of Prosthodontics, Farooqia Dental College and Hospital, Mysore, Karnataka 570015, India; ^4^Pandit Deendayal Dental College Hospital & Research Centre, Solapur, Maharashtra 413255, India

## Abstract

Mutilation of a portion of a face can cause a heavy impact on the self-image and personality of an individual. Acceptable cosmetic results usually can be obtained with a facial prosthesis. This paper describes prosthetic rehabilitation of a 60-year-old male patient having a left ocular defect. A technique to fabricate heat polymerizing polymethyl methacrylate was illustrated. The resultant prosthesis was structurally durable and aesthetically acceptable with satisfactory retention. The importance of meticulous treatment planning to tackle the challenges faced in fabricating an ocular prosthesis is explained with the relevant literature.

## 1. Introduction

Eyes are generally the first feature of the face to be noticed. Eye is a vital organ not only in terms of vision but also being an important component of facial expression. Loss of eye has a psychological effect on the patient. So a prosthesis should be provided as soon as possible for the psychological wellbeing of the patient [1]. An ocular prosthesis can be either ready-made (stock) or custom-made. Stock prosthesis comes in standard sizes, shapes, and colors. They can be used for interim or postoperative purposes [2–5]. Custom eyes have several advantages including better eyelid movements; even distribution of pressure due to equal movement thereby reducing the incidence of ulceration, improved fit, comfort, and adaptation improved facial contours, and enhanced esthetics gained from the control over the size of the iris, pupil and color of the iris and sclera [6–8]. Before starting the design of the prosthesis, it is essential to assess the psychological component in order to gain the confidence of the patient, in addition to a detailed medical history that includes the condition that led to the excision and enucleation in order to alert the possibility of recurrence [9].

## 2. Case Report

**Figure 1:**
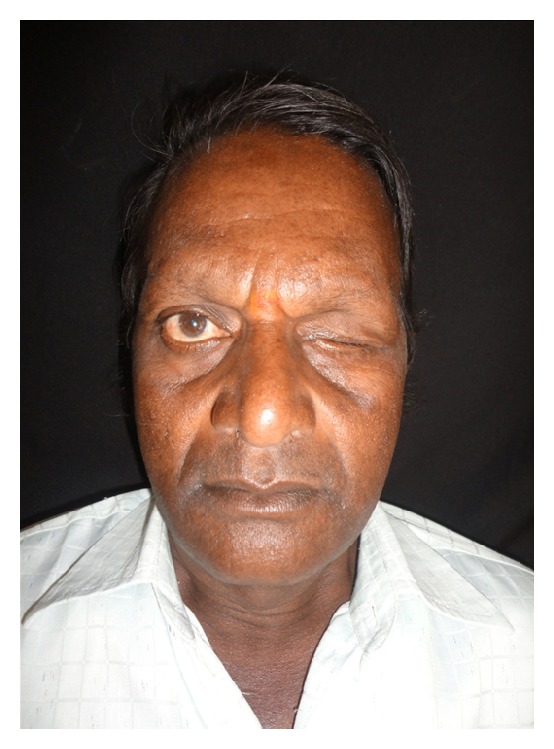
Patient with left side ocular defect.

**Figure 2:**
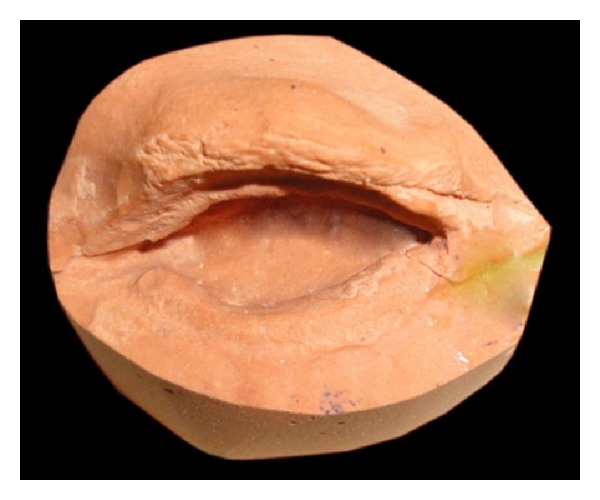
Two-piece dental cast.

**Figure 3:**
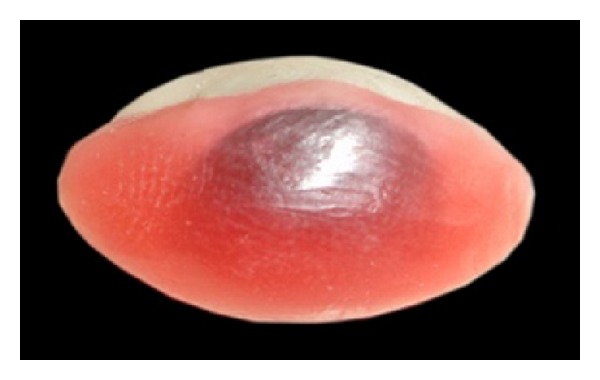
Thin layer of wax on sclera for the application of clear acrylic resin.

**Figure 4:**
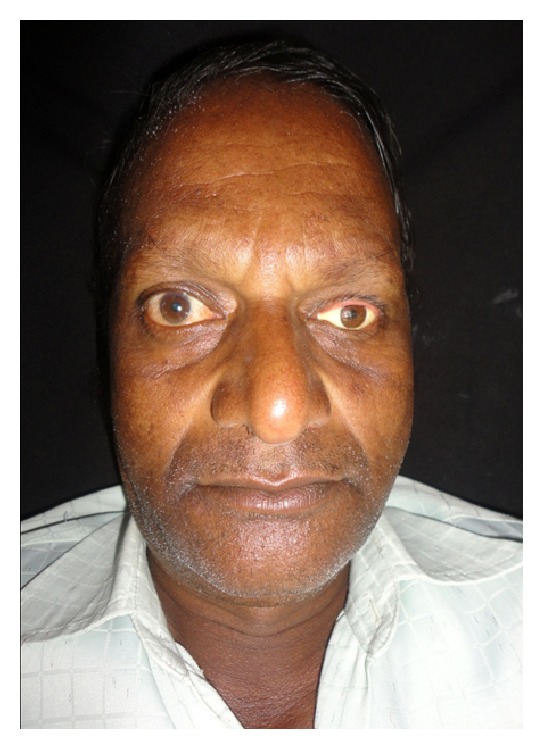
Ocular prosthesis inserted in the patients' eye.

A 60-year-old male patient reported to department of prosthodontics, HKE's S N Institute of Dental Sciences, Gulbarga, Karnataka, with a defect in the left eye. Case history revealed that he got his left eye enucleated when he was 25 years old due to a traumatic injury (Figure 1). On examination mucosa was healthy. Sulcus depth was sufficient enough to retain the restoration. A custom-made ocular prosthesis was planned to meet the needs of the patient since it would result in better esthetics than a stock eye shell. Petroleum jelly was applied to the eyebrows and skin around to prevent impression material from sticking to eyelashes. Primary impression was made with irreversible hydrocolloid material (Alginate, Prime Dental Products Pvt. Ltd., Mumbai, India). A cast was made from type II gypsum on which a special tray was fabricated using self-cure acrylic (Dental Products of India, Mumbai, India). A syringe was attached to the special tray through a perforation made at the centre of it. Impression of the defect was recorded using polyvinyl siloxane light viscosity material (Dentsply, Germany). Material was injected into the socket. The patient was instructed to make various eye movements as the material was injected so that the impression was recorded in the functional form. After the material had set, impression was retrieved from the socket and checked to ensure that all the surfaces were recorded. A two-piece type III dental stone cast was poured to immerse the lower part of the impression (Figure 2). After the stone had set, separating media was applied on the surface. Then a second layer was poured. Marking was made on all the four sides of cast for proper reorientation of the cast. Next, the wax pattern was fabricated by pouring the molten wax into the impression. The wax was properly contoured and carved to give it a simulation of the lost eye. The wax pattern was tried in patient's socket and checked for size, comfort, support, fullness, and retention by performing the functional movements. The wax pattern was flasked, dewaxed, and packed with tooth colored heat cure acrylic resin (Dental products of India, Mumbai), the shade of which was initially matched with the scleral portion of contralateral eye. Curing and polishing of scleral shell were done. Patient was made to sit upright and was asked to look straight with head erect. A second try in using custom made shell was done to mark the iris and corneal portion on the shell using contralateral iris and cornea as a reference. The size and color of cornea and iris portion were selected using prefabricated eye shell. It was trimmed to the desired size, which was previously marked on the shell during second try in. Acrylic was trimmed to a depth sufficient enough to incorporate the corneal portion which was retained using the same shade self-cure acrylic resin. Then a thin layer of wax was placed over the surface of scleral shell to create a space for clear acrylic, which gave a life-like effect (Figure 3). Flasking, dewaxing, packing, and curing of scleral shell were done using heat cure clear acrylic resin (Dental products of India, Mumbai) and small red colored silk thread. After curing, the prosthesis was finished and polished. Next, it was inserted in patient's eye (Figure 4).

## 3. Discussion

The ocular prosthesis is an artificial replacement for the bulb of the eye. After the surgeon enucleates the eye, prosthodontist is a person who comes into an act of providing the patient with an artificial eye to overcome the agony of losing an eye. A well-made and properly made ocular prosthesis maintains its orientation when patient performs various movements [10]. Now with the advent of newer materials like heat cure acrylic resin (DPI) as used here, it is possible to fabricate prosthesis with a life-like appearance. Moreover the use of stock ocular prosthesis of appropriate size and color cannot be neglected; a custom-made ocular prosthesis provide better results functionally as well as esthetically. It retains shape of defective socket, prevents collapse of lids, provides muscular functions of the lids, maintains palpebral opening, and gives a gaze similar to that of natural eye [10].

## 4. Conclusion

The esthetic outcome of the custom-made prosthesis was far better than the stock ocular prosthesis. The procedure used here is simple and cost effective. Although the patient cannot see by this prosthesis, this prosthesis will increase the self-confidence of the patient to face the world.
